# Unmasking *Borrelia* species: A comprehensive review of their presence in Iran

**DOI:** 10.1016/j.ijppaw.2025.101123

**Published:** 2025-07-29

**Authors:** Parisa Soltan-Alinejad, Mahmood Nikbakhtzadeh, Eslam Moradi-Asl

**Affiliations:** a Arthropod-Borne Diseases Research Center, Ardabil University of Medical Sciences, Ardabil, Iran; b Department of Health Sciences and Human Ecology, California State University, San Bernardino, USA

**Keywords:** *Borrelia*, Tick, Tick-borne disease, Lyme disease, Relapsing fever, Iran

## Abstract

*Borrelia* species are the causative agents of Lyme disease (LD) and tick-borne relapsing fever (TBRF) in humans and animals. These pathogens are transmitted through hard and soft ticks. The increasing tick population, influenced by climate change, underscores the urgent need for enhanced research on tick-borne diseases.

Iran, situated in southwestern Asia, boasts a diverse climate that supports a wide range of tick species and their vertebrate hosts. While TBRF is endemic to Iran, recent reports suggest the presence of LD in the country as well. Understanding the various *Borrelia* species, their tick vectors, human cases, affected reservoirs, and geographical distribution is crucial for assessing the epidemiology of TBRF and LD in Iran.

This comprehensive review examines the epidemiological patterns, geographical distribution, detection methods for these pathogens, providing critical insights into their public health significance.

## Highlights

• Tick borne relapsing fever (TBRF) is endemic in Iran, and Lyme disease (LD) has recently been reported.
• The distribution of *Borrelia* in Iran has increased, affecting 20 provinces.
• Mazandaran province shows the highest diversity of Borrelia species, with six types documented.
• Hard ticks (*I. ricinus* and *Rh. annulatus*) can also transmit TBRF in Iran, caused by *B. miyamotoi* and *B. theileri.*

## Graphical abstract

**Figure:**
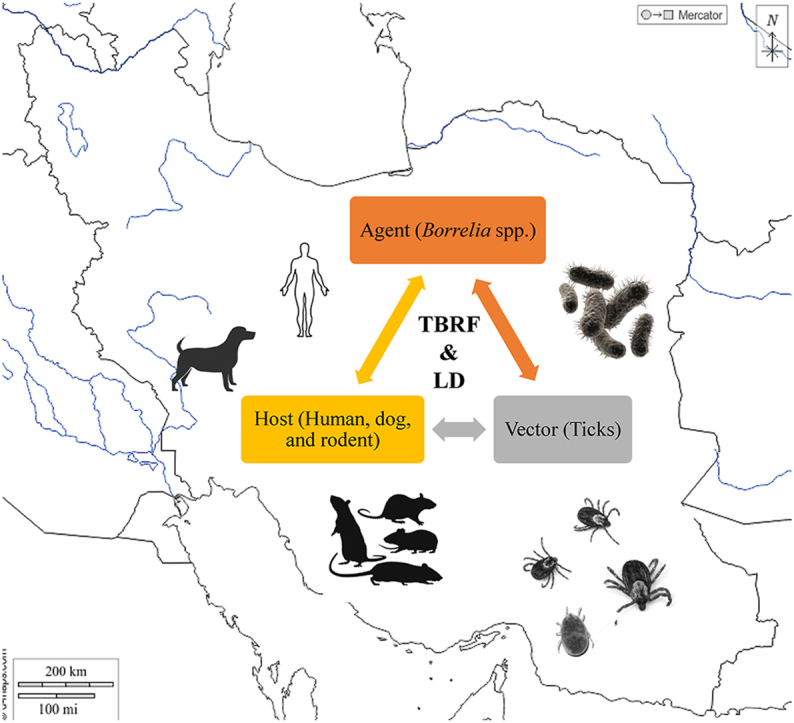

## 1 Introduction

*Borrelia* is classified within the spirochaetia class, comprising organisms that belong to the Spirochaetota phylum within the eubacteria kingdom. The genus *Borrelia* was recognized over a century after the discovery of *Borrelia recurrentis*, the pathogen responsible for louse-borne relapsing fever (LBRF) (Saint Girons et al., 1994). Various *Borrelia* species can cause diseases, including Relapsing Fever (Jakab et al., 2022) and Lyme Disease (LD) (Stanek and Strle, 2003). Relapsing fever is classified as either tick-borne (TBRF) or louse-borne (LBRF) (Felsenfeld,1971). LBRF is an anthroponotic disease caused by *B. recurrentis*, whereas TBRF is a zoonotic disease resulting from various *Borrelia* species (Jakab et al., 2022). *Borrelia* spp. are transmitted through both hard and soft ticks, as well as human body lice (Koutantou et al., 2024). Relapsing fever, encompassing both LBRF and TBRF, is typically observed in the western United States, Africa, Asia, Saudi Arabia, and Spain. These diseases remain epidemic in developing countries due to poverty, war, and famine (Hoch et al., 2015). The TBRF enzootic cycles differ, encompassing various tick species and their hosts, predominantly small rodents. Furthermore, they can infect humans, who act as accidental dead-end hosts (Killilea et al., 2008; Rodino et al., 2020). TBRF has recently been associated with travel-related infections following visits to endemic regions (Rebaudet and Parola, 2006). The global reduction of lice-infested populations has led to LBRF becoming an uncommon disease, while TBRF has become a growing concern in recent years (Jakab et al., 2022) (see Table 1, Table 2).

**Table 1:** Summary of published identified and unidentified *Borrelia* species in hosts across Iran.

	Host		*Borrelia* species	Location	Clinical complication	Laboratory method/Target gene	Accession Numbers	Treatment	Ref.
Identified *Borrelia* species responsible for TBRF	Accidental dead-end host	Human	*B. balthazardi*	Ardabil	–	Microscopic examination	–	–	Karimi et al., 1979
		Human/7-day-old neonate/Male (Congenital TBRF)	*B. persica*	Zanjan	Malaise, jaundice, weakness of neonatal reflexes, and sepsis	Microscopic examination	–	Erythromycin	Mahram and Ghavami, 2009
		Human/5 cases	*B. persica*	Khorasan-e Razavi	Relapsing episodes of fever, chills, and headache	Microscopic examination	–	–	Shayeghi et al., 2016
		Two-week-old puppy	*B. persica*	Tehran	Vomiting, diarrhea, and loss of appetite	Microscopic examination and PCR/*IGS*	KR816159	Metoclopramide, Vitamin B12, and Ampicillin	Shirani et al., 2016
	Reservoir	*Meriones persicus*	*B. duttonii-like spirochetes*	East Azerbaijan	–	Microscopic examination, qPCR, and PCR/*flaB, glpQ, groEL, p66, rrs, IGS*, and 16S rRNA	MW767941-5 for IGS, MW795343-6 for P66, MW79533-5 for flaB, MW737426-9 for rrs, MW795340-2 for groEL, and MW79536-9 for glpQ.	–	Ghasemi et al., 2021
		*M. persicus*	*B. duttonii*	East Azerbaijan	–	qPCR/16S rRNA	–	–	Rezaie et al., 2025
		*M. persicus*	*B. persica*	Hamadan	–	qPCR/16S rRNA	–	–	
Identified *Borrelia* species responsible for LD	Accidental dead-end host	Human/I case	*B. burgdorferi*	Mazandaran	Erythema migrans	ELISA	–	Doxycycline	Adabi et al., 2004
		Dogs	*B. burgdorferi*	Gilan, Mazandaran, and Golestan	No clinical signs	ELISA and Western blot	–	–	Hanifeh et al., 2012
		Companion dogs	*B. burgdorferi*	Ahvaz	No clinical signs	Immunochromatography assay (ICA)	–	–	Mosallanejad et al., 2015
		Dogs	*B. burgdorferi*	Ahvaz	–	ELISA	–	–	Rezaei et al., 2016
		Companion dogs	*B. burgdorferi*	Fars	No clinical signs	ELISA	–	–	Esmailnejad et al., 2017
		Human/1 case (neuroborreliosis)	*B. burgdorferi*	–	Seizures	Chestcomputed tomography (CT) scan, MRI, and serological tests	–	Ceftriaxone	Sayad et al., 2023
		Guard dogs	*B. burgdorferi*	Isfahan	No clinical signs	Microscopic examination and PCR/16S rRNA	–	–	Chaleshtory et al., 2023
Unidentified *Borrelia* species	Human/13 cases		*Borrelia* spp.	Kazeroun	–	Microscopic examination	–	–	Janbakhsh and Ardelan, 1977
	Human/391 cases		*Borrelia* spp.	Ardabil	Headache, fever, chills, nausea, vomiting, abdominal pain, cough, sweating, hematuria, jaundice, arthralgia, petechiae, photophobia, and eosinophilia	Microscopic examination	–	–	Arshi et al., 2002
	Human/1 case		*Borrelia* spp.	Ardabil	Headache, vomiting, fever, positive Kernig's sign, and neck stiffness (Meningitis)	Microscopic examination	–	Doxycycline	Majid-Pour, 2003
	Borrelia meningitis								
	Human/97 cases		*Borrelia* spp.	Kurdistan	Sweats, nervousness, headache, fever, chills, vomiting, stomach ache, myalgias, nose bleeding, cough, arthralgia, and photophobia	Microscopic examination	–	–	Rafinejad et al., 2012
	Human/138 cases		*Borrelia* spp.	Kurdistan	Chills, fever, sweating, headache, abdominal pain, vomiting, myalgia, arthralgia, nervousness, cough, photophobia and nose bleeding	Microscopic examination	–	–	Kassiri et al., 2014b
	Human/148 cases		*Borrelia* spp.	Kurdistan	Fever, chills and headache	Microscopic examination	–	–	Moemenbellah-Fard et al., 2009
	Human/11 cases		*Borrelia* spp.	Kurdistan	Chills, fever, sweating, headache, abdominal pain, vomiting, myalgia, arthralgia, nervousness, cough, photophobia and nose bleeding	Microscopic examination	–	–	Kassiri et al., 2014a
	Human/1 case		*Borrelia* spp.	Fars	Fever and tachycardia	Microscopic examination	–	Intravenous penicillin G four times a day for 24 h and Oral penicillin for 10 days.	Pouladfar et al., 2008
	Human/14 cases		*Borrelia* spp.	Hormozgan	Headache, fever, chills, nausea, vomiting, abdominal pain, cough, sweating, hematuria, jaundice, arthralgia, petechiae, photophobia, eosinophilia, muscle and joint pain	Microscopic examination/PCR	–	Tetracycline	Naddaf et al., 2015
	One-month-old puppy		*Borrelia* spp.	Tehran	Icteric mucous membranes, malaise, tenderness upon palpation, pustular lesions, loss of appetite, diarrhea, and fever	Microscopic examination	–	Prednisolone and Doxycycline	Rostami et al., 2011
	Human/276 cases		*Borrelia* spp.	Hamedan	–	Microscopic examination	–	–	Nazari and Najafi, 2016
	Human/2 cases		*Borrelia* spp.	Jask/Hormozgan	Fever, fatigue, and headache	PCR		Tetracycline	Naddaf et al., 2017
	Human/1 case		*Borrelia* spp.	Tehran	Joint pain in the left elbow and left ankle, erythema migrans on foot	Microscopic examination, ELISA, and Western blot		Doxycycline	Siadati, 2006
	Sheep and goats/1018 cases		*Borrelia* spp.	West Azerbaijan	–	PCR/*glpQ*, *IGS*, and *flaB*	KX683867 for *IGS*, KX683864 and KX683865 for *glpQ*, and KX683866 for *flaB*	–	Enferadi et al., 2024a
	Cats and dogs		*Borrelia* spp.	West Azerbaijan	No clinical signs	PCR/*5–23 S* rRNA	OR770094, OR770195, OR770198, and OR771914	–	Ownagh et al., 2024
	Hares and long-eared hedgehogs (*Hemiechinus megalopolis*)		*Borrelia* spp.	Sistan and Baluchistan	–	PCR/*5–23S* rRNA	OR400934 and OR398185	–	Sarani et al., 2024

**Table 2:** Summary of published identified and unidentified *Borrelia* species in different tick species in Iran**.**

Disease	Tick vector	Tick status in the field (Feeding or questing)	*Borrelia* species	Province	Laboratory method/Target gene	Accession Numbers	Ref.
Identified *Borrelia* species related to TBRF	*O. tholozani*	–	*B. persica*	Markazi	–	**-**	Moradi-Asl and Jafari, 2020
	*O. tholozani*	Questing and feeding on domestic animals	*B. persica*	Semnan	Animal inoculation, microscopic examination	–	Nekoui et al., 1999
	*O. tartakovskyi*	Questing in rodent burrows and bird nests	*B. latyschewii*	Khorasan-e Razavi	Animal inoculation, microscopic examination	–	Piazak et al., 2000
	*O. tholozani*	Questing in human dwelling and animal	*B. persica*	Hamedan	Animal inoculation, microscopic examination	–	Vatandoost et al., 2003
	*O. tholozani*	Questing in human dwelling, animal shelters	*B. persica*	Kurdistan	Animal inoculation, microscopic examination	–	Rafinejad et al., 2012
	*O. tholozani*	Questing in human dwelling and animal	*B. persica*	Qazvin	Animal inoculation, microscopic examination	–	Aghighi et al., 2007
	*O. erraticus*		*B. microti*			–	
	*O. tholozani*	Questing in human dwelling, animal shelters, and rodent burrows	*B. persica*	Qazvin	PCR-RFLP/16S rRNA	EU914141	Barmaki et al., 2010
	*O. erraticus*	Feeding on rodents	*B. microti*	Alborz	Animal inoculation, microscopic examination, and PCR/16S rRNA, *flaB*, *glpQ,* and *IGS*	JF803950, JF825472, JF825473, and JQ436580	Naddaf et al., 2012
	*O. tholozani*	Questing in human dwelling, animal shelters	*B. persica*	Khorasan-e Razavi	Animal inoculation, microscopic examination	–	Shayeghi et al., 2016
	*A. persicus*	Questing in cracks of aviary	*B. anserina*	Lorestan	PCR/*flaB*	KY438930	Chegeni et al., 2017
	*I. ricinus*	Questing and feeding (camels, goats, donkeys, hedgehogs, sheep, rodents, horses, cattle, and dogs)	*B. miyamotoi*	Mazandaran	qPCR and PCR/16S rRNA, *flaB,* and *rrs-rrlA*	MN958345 to MN958348	Naddaf et al., 2020
	*Rh. annulatus*	Questing and feeding (dogs, sheep, cattle, and goats)	*B. theileri*	Mazandaran	qPCR and PCR/16S rRNA, *flaB,* and *glpQ*	OR037296-OR037302 for *flaB* and OR037292- OR037295 for *glpQ*	Milani et al., 2024
Identified *Borrelia* species related to LD	*I. ricinus*	Questing and feeding (camels, goats, donkeys, hedgehogs, sheep, rodents, horses, cattle, and dogs)	*B. bavariensis, B. valaisiana, B. afzelii*	Mazandaran	qPCR and PCR/16S rRNA, *flaB,* and *rrs-rrlA*	MN958342, MN958343, MN958344	Naddaf et al., 2020
	*I. ricinus*		*B. garinii*	Gilan		MN958341	
Unidentified *Borrelia* species	*O. tholozani*	Questing in human dwelling, animal shelters, and rodent burrows	*Borrelia* spp.	Kurdistan	Animal inoculation, microscopic examination	–	Moemenbellah-Fard et al., 2009
	*O.lahorensis, O.tholozani, and A. persicus*	Questing	*Borrelia* spp.	Ardabil	Animal inoculation, microscopic examination	–	Arshi et al., 2002
	*O. lahorensis*	Questing in human dwelling, animal shelters, and rodent burrows	*Borrelia* spp.	Takistan/Qazvin	PCR-RFLP/16S rRNA	–	Barmaki et al., 2010
	*O. tholozani*	Questing in animal shelters	*Q*	Zanjan	Animal inoculation, microscopic examination	–	Mohammadi et al., 2013
	*O. tholozani/*eggs	Questing in animal dwelling	*Borrelia* spp.	Ardabil	Animal inoculation, microscopic examination and PCR/*rrs-rrlA*	–	Aghaei et al., 2014
	*Rh. turanicus, and Rh. sanguineus*	Questing and feeding (camels, goats, donkeys, hedgehogs, sheep, rodents, horses, cattle, and dogs)	*Borrelia* spp.	Golestan	qPCR and PCR/16S rRNA, *flaB,* and *rrs-rrlA*	MN958349 to MN958351	Naddaf et al., 2020
	*Rh. sanguineus, H. asiaticumss, H.aegyptium, H. anatolicum*	Feeding on sheep and goats	*Borrelia* spp.	West Azerbaijan	PCR/16srRNA*, 5S-23SrRNA,* and *ospA*	OQ073805.1, OR342388.1, OR352151.1, and OR345451.1.	Enferadi et al., 2024b
	*Hyalomma aegyptium. and Rh. sanguineus*	Feeding on rabbits and hedgehogs.	*Borrelia* spp.	Sistan and Baluchistan	PCR/*5–23S* rRNA	–	Sarani et al., 2024

LD is recognized as the most rapidly increasing tick-borne zoonotic disease worldwide, with reported cases in more than 60 countries and established endemic regions in North America, Europe, and Asia (Donohoe et al., 2015). It is transmitted through infected hard tick bites, primarily from ticks of the *Ixodes* genus (Giesen et al., 2024). The reservoir hosts for LD include various mammalian species, particularly rodents, as well as other animals capable of hosting hard ticks (Kazemi-Moghaddam et al., 2019). Between 1982 and 2022, the Centers for Disease Control and Prevention (CDC) documented more than 844,000 cases of LD (Mead et al., 2024). The burden of LD has increased, spreading to previously non-endemic regions (Stone et al., 2017). In this study, we assessed the status of *Borrelia* spp. in Iran, located in southwestern Asia.

## 2 Epidemiological pattern of identified *Borrelia* species

### 2.1 TBRF history, vectors, and hosts (reservoir and dead-end)

TBRF is recognized as an endemic disease in Iran. Between 1997 and 2006, more than 1400 cases were documented across 19 provinces (Asl et al., 2009). The epidemiology of the disease in various regions is influenced by the interactions between ticks, *Borrelia* species, and environmental conditions within their respective distribution areas (Vatandoost et al., 2003). Previously, *B. persica* was identified as a causative agent, with *Ornithodoros tholozani* reported as the main vector in Iran (Karimi et al., 1979; Naddaf et al., 2015). Additionally, *B. baltazardi* and *B. latyschewii* have been recorded in northwestern and northeastern Iran (Karimi et al., 1979). Concerning TBRF within their natural enzootic cycles, both humans and dogs serve as accidental dead-end hosts (Rodino et al., 2020). An exception to this is *B. duttonii*, for which humans might act as the reservoir (Jakab et al., 2022). *B. persica* has been documented in human TBRF cases in the provinces of Zanjan and Khorasan-e-Razavi (Mahram and Ghavami, 2009; Shayeghi et al., 2016). *B. baltazardi* has been detected in the blood serum of patients in Ardabil (Karimi et al., 1979). TBRF can manifest in neonates, including cases of congenital disease. A congenital TBRF case has been documented in Zanjan, where a 7-day-old neonate exhibited malaise, jaundice, diminished neonatal reflexes, and sepsis, but lacked hepatosplenomegaly**,** fever, and respiratory distress (Mahram and Ghavami, 2009). However, infected human cases in Khorasan-e-Razavi presented with relapsing fever episodes, chills, and headaches (Shayeghi et al., 2016). An instance of *B. persica* infection has also been documented in a two-week-old puppy in Tehran, which exhibited vomiting, diarrhea, and loss of appetite (Shirani et al., 2016). Wild rodents are generally the primary reservoir hosts (Rebaudet and Parola,2006). The rodent species *Meriones persicus*, commonly found in Iran, serves as a host for *Borrelia* species responsible for TBRF. In the provinces of East Azerbaijan and Hamedan, *B. persica* and *B. duttonii*, as well as *B. duttonii*-like spirochetes, have been successfully isolated from *M. persicus* (Adabi et al., 2004; Ghasemi et al., 2021; Rezaie et al., 2025).

Information on TBRF tick vectors indicates that *B. persica* has been identified in *O. tholozani* across the provinces of Markazi, Semnan, Hamedan, Kurdistan, Qazvin, and Khorasan-e- Razavi (Nekoui et al., 1999; Vatandoost et al., 2003; Aghighi et al., 2007; Barmaki et al., 2010; Rafinejad et al., 2012; Shayeghi et al., 2016; Moradi-Asl,2020). *B. microti* has been reported from *O. erraticus* in Qazvin and Alborz (Aghighi et al., 2007; Naddaf et al., 2012). Other studies have recorded *B. anserine* and *B. latyschewii* from *Argas persicus* and *O. tartakovsky*, respectively, in Lorestan and Khorasan-e-Razavi (Piazak et al., 2000; Chegeni et al., 2017).

Recent findings indicate that hard ticks can also transmit TBRF. *Ixodes ricinus* and *Rhipicephalus annulatus* have been documented as TBRF hard tick vectors for *B. miyamotoi* and *B. theileri* in Mazandaran (Naddaf et al., 2020; Milani et al., 2024).

### 2.2 LD history, vectors, and hosts (reservoir and dead-end)

**Fig. 1:**
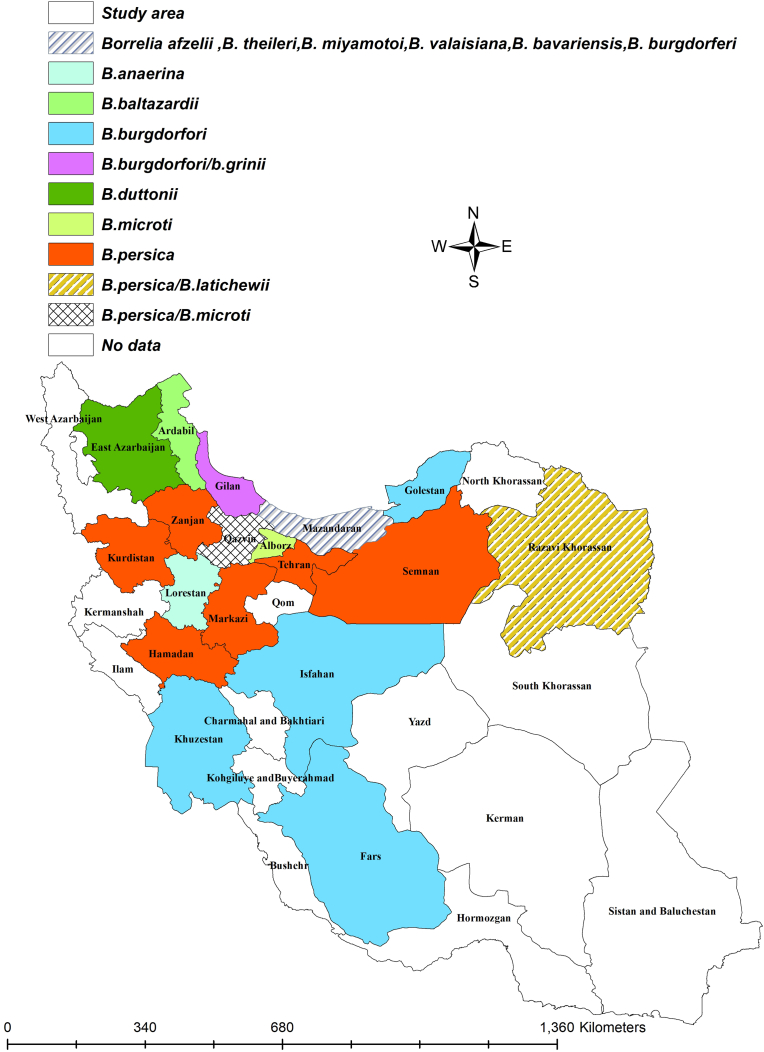
Different types of *Borrelia* species by the counties.

The first report of endemic LD caused by *B. burgdorferi* in Iran occurred in 1997 (Chams-Davatchi, 1997), serving as an alert for medical professionals to consider LD in their diagnostic evaluations. Following this case report, several LD patients have been documented over time in the country. All identified *Borrelia* species in Iran are shown in Fig. 1. So far, among the various *Borrelia* species that cause LD, only *B. burgdorferi* has been reported from hosts and ticks in Iran. Humans and dogs inadvertently become part of this cycle, acting as dead-end hosts (Humair and Gern, 2000; Wagner and Erb, 2012). Conversely, small wild mammals such as rodents and possibly some migratory birds play a role as reservoirs in maintaining the Lyme borreliosis cycle (Wagner and Erb, 2012). In Iran, there have been two reported cases of human Lyme borreliosis. One case occurred in Mazandaran (Adabi et al., 2004), while the region associated with the Lyme neuroborreliosis case has not been specified (Sayad et al., 2023). In Lyme neuroborreliosis, initially, the patient exhibited low-grade fever, headache, vomiting, and malaise; however, after a week, they were hospitalized due to the onset of seizures (Sayad et al., 2023). This zoonotic disease has also been documented in dogs across various regions of Iran, including Gilan, Mazandaran, Golestan, Khuzestan, Fars, and Isfahan (Hanifeh et al., 2012; Mosallanejad et al., 2015; Rezaei and Gh, 2016; Esmailnejad et al., 2017; Chaleshtory et al., 2023). Notably, *B. burgdorferi* did not cause any clinical signs in dogs (Hanifeh et al., 2012; Mosallanejad et al., 2015; Esmailnejad et al., 2017; Chaleshtory et al., 2023). Findings regarding tick vectors of LD indicated that *B. bavariensis*, *B. valaisiana*, *B. afzelii*, and *B. garinii* have been successfully isolated from *I. ricinus* in Mazandaran and Gilan provinces (Naddaf et al., 2020).

**Fig. 2:**
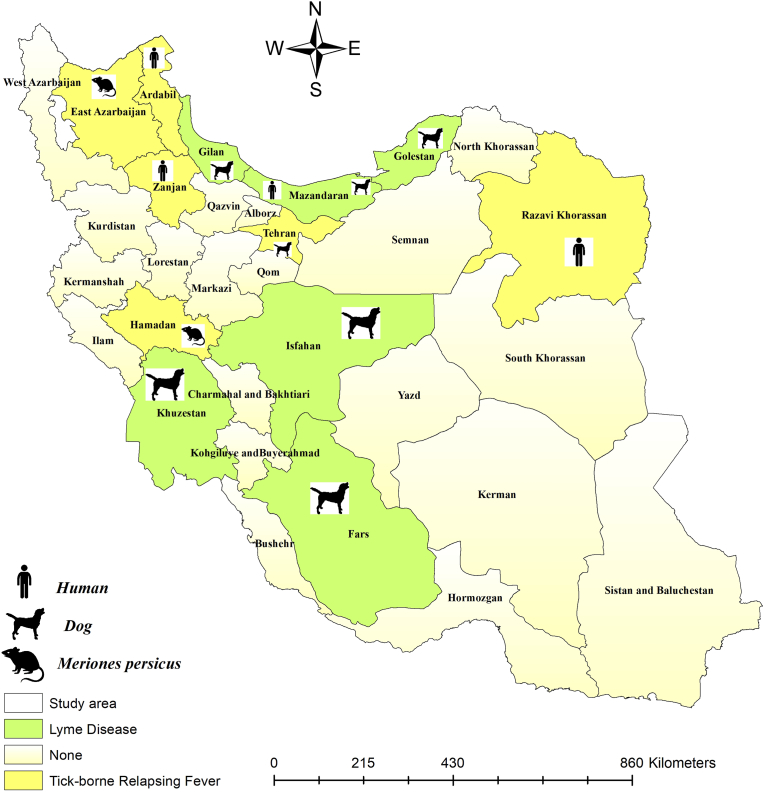
Identified *Borrelia* species in various hosts by the counties. The symbols represent humans, dogs, and rodents, indicating the *Borrelia*-infected specimens.

**Fig. 3:**
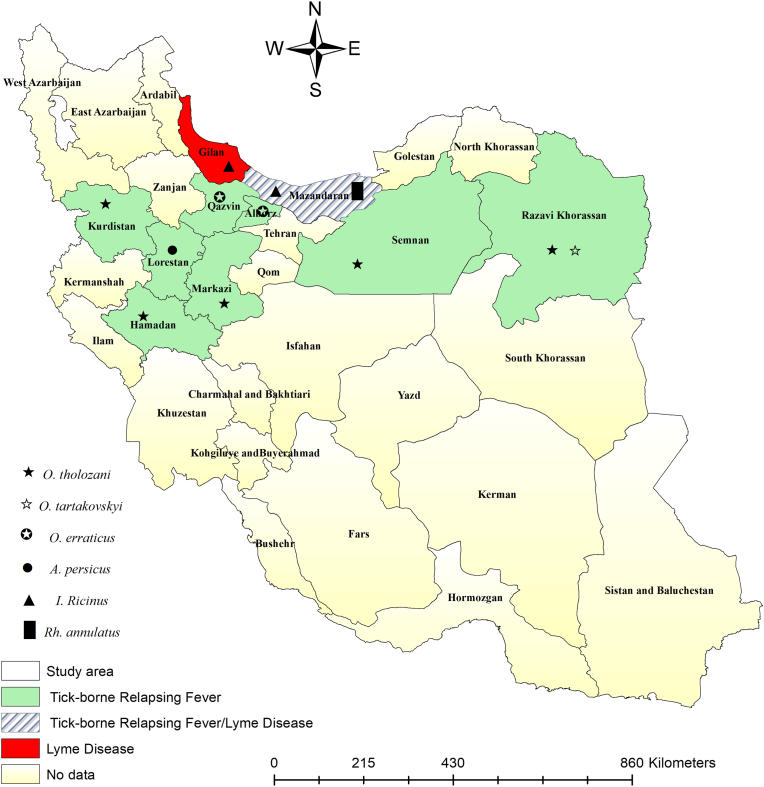
Identified *Borrelia* species in various tick species by the counties. The symbols represent different species of ticks infected with *Borrelia*.

The geographical distribution of identified *Borrelia* species in hosts is presented in Fig. 2. Additionally, the geographical distribution of identified *Borrelia* species from different tick species is shown in Fig. 3.

### 2.3 Unidentified *Borrelia* species reported in vectors and hosts

**Fig. 4:**
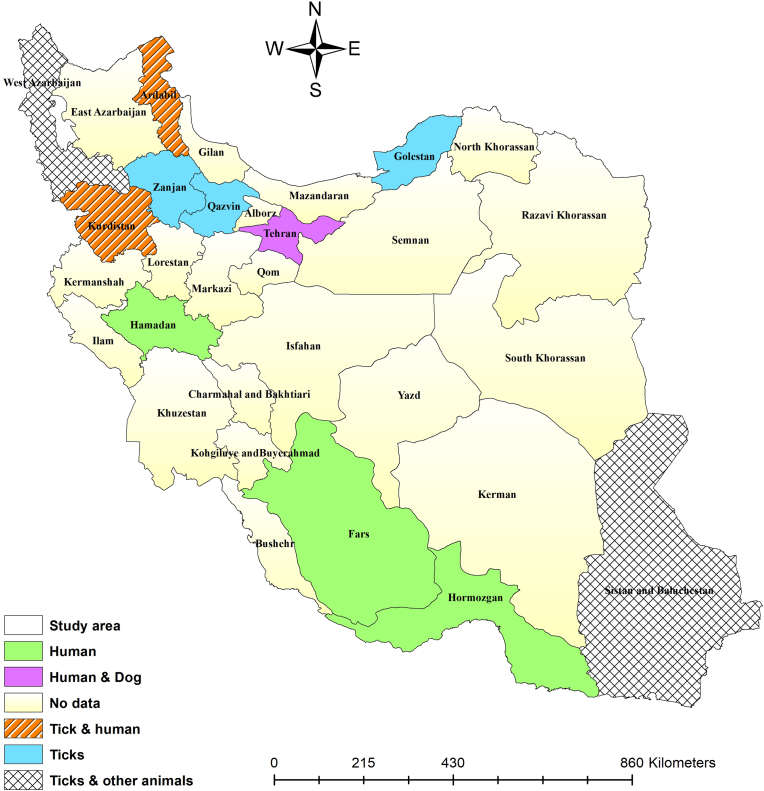
Unidentified *Borrelia* species in various hosts and tick species by the counties.

Previous studies have recorded human cases of TBRF symptoms in Fars (Janbakhsh and Ardelan, 1977), Ardabil (Arshi et al., 2002; Majid-Pour, 2003), Kurdistan (Moemenbellah-Fard et al., 2009; Rafinejad et al., 2012; Kassiri et al., 2014; Kassiri et al., 2014), Fars (Pouladfar et al., 2008), Hormozgan (Naddaf et al., 2015, Naddaf et al., 2017), and Hamedan Provinces (Nazari and Najafi, 2016). However, the species of *Borrelia* involved in these cases have not been identified. A human case with LD clinical symptoms was reported from Tehran, while the type of *Borrelia* is unknown (Siadati, 2006). A one-month-old puppy was diagnosed with a *Borrelia* spp. infection in Tehran (Rostami et al., 2011). Blood samples from cats, dogs, sheep, and goats in West Azerbaijan (Enferadi et al., 2024; Ownagh et al., 2024), and hares and long-eared hedgehogs (*Hemiechinus megalotis*) in Sistan and Baluchistan revealed the presence of *Borrelia* spp. (Sarani et al., 2024). Unidentified *Borrelia* species have also been documented in soft and hard tick vectors. The identified soft ticks were infected with *Borrelia* spp. include *O. tholozani* from Kurdistan (Moemenbellah-Fard et al., 2009), Ardabil (Arshi et al., 2002; Aghaei et al., 2014), and Zanjan (Mohammadi et al., 2013); *O. lahorensis* from Ardabil (Arshi et al., 2002) and Qazvin (Barmaki et al., 2010); and *A. persicus* from Ardabil (Arshi et al., 2002). The identified hard ticks are infected with *Borrelia* spp. include *Rh. turanicus* and *Rh. sanguineus* from Golestan (Naddaf, Mahmoudi et al.,2020); *Rh. sanguineus, Hyalomma asiaticum, H. aegyptium,* and *H. anatolicum* from West Azerbaijan (Enferadi et al., 2023); and *Hyalomma aegyptium. and Rh. sanguineus* from Sistan and Baluchistan (Sarani et al., 2024). The geographical distribution of unidentified *Borrelia* species in hosts and ticks has been illustrated in Fig. 4.

## 3 Laboratory methods for *Borrelia* detection

### 3.1 Microscopic examination

Giemsa staining was employed for the identification of *Borrelia* spp. in the microscopic method. The stained slide demonstrated the presence of spirochetes within the thin blood smear obtained from an infected canine (Rostami et al., 2011; Shirani et al., 2016; Chaleshtory et al., 2023), *M. persicus* (Ghasemi et al., 2021), and human (Janbakhsh and Ardelan, 1977; Arshi et al., 2002; Majid-Pour, 2003, Siadati, 2006; Pouladfar et al., 2008; Mahram and Ghavami, 2009; Moemenbellah-Fard et al., 2009; Rafinejad et al., 2012; Kassiri et al., 2014; Kassiri et al., 2014; Naddaf et al., 2015; Nazari and Najafi, 2016; Shayeghi et al., 2016).

### 3.2 Animal inoculation

It is important to understand that the sensitivity of microscopy is defined by specific positivity thresholds, which are estimated to be *10*^*5*^ spirochetes per mL of blood for thin smears and *10*^*4*^ spirochetes per mL for thick smears (Hovette et al., 2001). As a result, more investigations use animal inoculation before direct microscopic examination. Bacteria can be introduced into the animal either by direct injection or by allowing ticks to feed on it. One or two weeks later, blood samples from the inoculated animals were tested for *Borrelia* using Giemsa staining, microscopic examination, or PCR. In this method, a suspension made from live ticks, including *O. lahorensis, O. tholozani*, and *A. persicus*, was injected into the peritoneum of guinea pigs and white mice in Ardabil for *Borrelia* spp. (Arshi et al., 2002). *O. tholozani* was inoculated into suckling Syrian hamsters or white mice for *Borrelia* spp. in Kurdistan (Moemenbellah-Fard et al., 2009). *O. tholozani* was also intraperitoneally inoculated into white mice with *B. persica* (Nekoui et al., 1999; Vatandoost et al., 2003; Mohammadi et al., 2013; Shayeghi et al., 2016), and *O. erraticus* was inoculated into guinea pigs with *B. microti* (Naddaf et al., 2012). In the tick-feeding method, unfed *O. tartakovskyi* infected with *B. latyschewii, and O. tholozani* infected with *B. persica* were fed on white mice in Khorasan-e-Razavi and Hamedan, respectively (Piazak et al., 2000, Vatandoost et al., 2003). In another study, infected *O. tholozani* were fed on guinea pigs, while *O. erraticus* were fed on newborn mice. As a result, *B. persica* and *B. microti* were successfully isolated from *O. tholozani* and *O. erraticus*, respectively (Aghighi et al., 2007). Unfed *O. tholozani* infected with *Borrelia* spp. fed on guinea pigs and mice in Semnan, Zanjan, and Ardabil (Nekoui et al., 1999; Arshi et al., 2002; Mohammadi et al., 2013). In a separate investigation conducted in Kurdistan, *O. tholozani* was examined for the presence of *Borrelia* spp. through a three-step process: the ticks were first crushed and inoculated into guinea pigs and white mice, followed by blood-feeding on the guinea pigs, and finally, a subcutaneous injection of the crushed ticks was administered (Rafinejad et al., 2012).

### 3.3 Molecular technique

The Nested-PCR technique was employed for the identification of *Borrelia* species after DNA extraction from tissues (tick and animal) or blood samples. This technique uses specific primers to precisely identify the species of spirochete. Multilocus sequence analysis (MLSA) used for *Borrelia* identification includes the intergenic spacer region (IGS) (Naddaf et al., 2012; Naddaf et al., 2015; Shirani et al., 2016; Naddaf et al., 2017; Ghasemi et al., 2021), 5–23S rRNA gene (Mosallanejad et al., 2015; Enferadi et al., 2023; Enferadi et al., 2024a; Ownagh et al., 2024; Sarani et al., 2024), non-coding *rrs-rrlA* spacer (Aghaei et al., 2014; Naddaf et al., 2020; Ghasemi et al., 2021), flagellin (*flaB*) (Chegeni et al., 2017; Naddaf et al., 2017; Naddaf et al., 2020; Ghasemi et al., 2021; Milani et al., 2024), glycerophosphodiester phosphodiesterase (*glpQ*) (Naddaf et al., 2012; Naddaf et al., 2017; Ghasemi et al., 2021; Milani et al., 2024), *groEL* (Ghasemi et al., 2021), *p66* (Ghasemi et al., 2021), 16S rRNA region (Chaleshtory et al., 2023), and *ospA* (Enferadi et al., 2023; Enferadi et al., 2024). In several investigations, the qPCR/Real-Time PCR technique was utilized to detect *Borrelia* spp. using a specific probe targeting the 16S rRNA region (Naddaf et al., 2020; Ghasemi et al., 2021; Milani et al., 2024; Moradkasani et al., 2024; Rezaie et al., 2025). Furthermore, PCR-restriction fragment length polymorphism (RFLP) analysis was employed to identify *B. persica* in ticks (Barmaki et al., 2010).

### 3.4 Serological tests

The serological testing for *Borrelia* species, particularly in LD, generally consists of a two-phase procedure. The initial phase employs an enzyme-linked immunosorbent assay (ELISA) to identify antibodies targeting *B. burgdorferi*. If the outcome of this first test is either positive or uncertain, a Western blot test is administered to confirm the diagnosis. In several studies, ELISA and/or Western blot, and immunochromatography assay (ICA) techniques were utilized to detect the presence of *B. burgdorferi* in dogs (Adabi et al., 2004; Siadati, 2006; Hanifeh et al., 2012; Mosallanejad et al., 2015; Rezaei and Gh,2016; Esmailnejad et al., 2017; Sayad et al., 2023).

## 4 The negative findings regarding *Borrelia* presence in hosts and ticks

During the period from 2011 to 2012, blood smear examinations of febrile patients in the city of Chabahar revealed no signs of spirochetes associated with *Borrelia* (Metanat et al., 2014). None of the avian blood samples collected in Lorestan Province showed any signs of infection with *B. anserina* (Chegeni et al., 2017). In Khorasan Province, PCR testing indicated that there was no evidence of *Borrelia* spp. infection among 66 patients suffering from morphoea symptoms (Yazdanpanah et al., 2015). Given that small mammals and rodents are considered reservoirs of TBRF and LD disease, it is important to note that, none of the rodents and small mammals in Mazandaran, Gilan, and Golestan Provinces, including *Rattus norvegicus, Microtus obscurus, Mus musculus castaneus, Rattus rattus, Apodemus hyrcanicus, Apodemus uralensis, Nesokia indica, Microtus paradoxus, Crocidura caspica, Crocidura suaveolens*, and *Erinaceus concolor*, exhibited evidence of *Borrelia* spp. presence within their internal organs (Naddaf et al., 2020). In a study involving *R. norvegicus* gathered from Tehran, antibodies (IgG) against *Borrelia* spp. were not found (Azimi et al., 2024). *Borrelia* spp. was not detected in dromedary camels (*Camelus dromedarius*) assessed in Fars, Kerman, and Sistan and Baluchistan Provinces (Sazmand et al., 2019; Heidari et al., 2023). An investigation was conducted to survey the presence of *Borrelia* spp. in Iran, focusing on ornamental birds across four provinces: Tehran, East Azerbaijan, Yazd, and Khuzestan. The results indicated that *Borrelia* was absent in all the birds that were studied (Enferadi et al., 2024). Regarding ticks, no *Borrelia* was found in these investigations: *A. persicus* in Qazvin and West Azerbaijan (Barmaki et al., 2010; Enferadi et al., 2024) and *I. ricinus* in Gilan, Mazandaran, and Golestan (Yousefi-Behzadi et al., 2022).

## 5 Discussion and conclusion

### 5.1 Identified *Borrelia* species regarding TBRF and LD

Our review study found that 20 provinces in Iran are affected by *Borrelia* species. The geographical distribution map indicates that B. persica and B. burgdorferi are the most prevalent *Borrelia* species in Iran, respectively. The causative agents of TBRF are chiefly present in the northern regions of Iran. In contrast, *B. burgdorferi* has been reported in northern provinces, as well as in central, southern, and southwestern regions of Iran. Mazandaran exhibits the highest diversity of *Borrelia*, where six species of the pathogen have been documented. This notable diversity may be attributed to the province's geographical location and the presence of migratory birds, which have a remarkable ability to host various animal species, especially migratory birds (Jakab et al., 2022), likely contributing to the spread of these pathogens. Moreover, the prolonged attachment and feeding period of hard ticks enhances their ability to migrate over vast distances (Spach et al., 1993; Guberman et al., 1994), facilitating the dissemination of *Borrelia* species.

### 5.2 TBRF vectors and hosts

Humans and dogs are reported as dead-end hosts, while *M. persicus* has been documented as a reservoir in Iran. Considering a documented neonatal case in an endemic region, neonatal borreliosis should be considered in any newborn presenting with symptoms indicative of neonatal sepsis. Additionally, any history of febrile illnesses experienced by the mother during pregnancy, particularly in the final days of gestation, is crucial to consider. For accurate diagnosis, peripheral blood smears from the infant must be obtained at least three times (Mahram and Ghavami, 2009). The transmission of TBRF in Iran is predominantly associated with four species of soft ticks, including *O. tholozani, O. erraticus, O. tartokovyskyi,* and *A. persicus*. Surprisingly, hard ticks such as *I. ricinus* and *Rh. annulatus* can also transmit TBRF(Naddaf et al., 2020; Milani et al., 2024). Climate change can increase the risk of TBRF by expanding the activity and range of ticks and increasing the number of susceptible hosts (Bouchard et al., 2019).

### 5.3 LD vectors and hosts

The known dead-end hosts of LD in Iran are humans and dogs. Although *B*. *burgdorferi* has been predominantly identified in canine populations, human cases have been reported exclusively in Mazandaran. The incidence of LD in animals has increased over the past several years, specifically from 2009 to 2023. Despite reports of LD cases in six provinces across the country, the investigation of hard tick vectors responsible for the disease have been conducted only in Mazandaran.

### 5.4 Laboratory methods for *Borrelia* detection

The diagnosis of TBRF and LD is based on a combination of clinical observations and laboratory tests, none of which alone can definitively confirm infection with *Borrelia* species (Mead et al., 2024). For TBRF, preparing a thin blood smear followed by microscopic examination is a standard diagnostic method. However, comparative studies have demonstrated that microscopic examination may fail to detect spirochetes in some cases, whereas molecular techniques such as PCR and quantitative PCR (qPCR) can successfully identify their presence (Siadati, 2006). In fact, qPCR has been shown to possess higher sensitivity than traditional PCR, particularly for detecting spirochete DNA at low cycle threshold (Ct) values (Gil et al., 2005; Naddaf et al., 2020; Milani et al., 2024). Therefore, molecular methods could be more reliable techniques for diagnosing TBRF. The use of serological testing for diagnosing TBRF is limited because high seroprevalence does not always indicate an active infection, delayed seroconversion can hinder timely treatment decisions, and assay cross-reactivity complicates distinguishing TBRF from Lyme disease (Magnarelli et al., 1987). In contrast, the diagnosis of LD is primarily depends on serological tests, including ELISA, Western blot, and ICA (Marques,2015).

## 6 Literature gap and future research

Review studies often highlight gaps in the literature and suggest future research. In this study, we aim to recommend effective field studies to improve understanding and control of tick-borne diseases in Iran. In East Azerbaijan, while *M. persicus* has been found infected with *B. duttonii*, ticks in this area have yet to be tested for *Borrelia* species. Given that infected humans serve as reservoir for *B. duttonii*, studying local tick populations in this province is crucial. Similarly, an animal case report in West Azerbaijan suggests the presence of *Borrelia*, but comprehensive investigation into *Borrelia* species and tick infection are still lacking. In Zanjan Province, the identification of *B. persica* in *O. tholozani* remains inconclusive and relies primarily on earlier reports, highlighting the need for updated molecular and phylogenetic studies. Notably, molecular investigations in Hormozgan involving patients with symptoms of TBRF have revealed diverse *Borrelia* species that cluster into distinct clades. A focused research project on these pathogens has the potential to uncover a novel *Borrelia* type in Iran. Nanopore-based metagenomic studies have demonstrated higher sensitivity in detecting a wide range of tick-borne viruses and bacteria, enabling the molecular characterization of novel viral and bacterial strains in cases where traditional methods were inadequate or unsuccessful (Ravi et al., 2019; Ergunay et al., 2023; Kipp et al., 2023). Furthermore, in central, southern, and southwestern Iran—regions where LD has been identified in dogs, dedicated studies assessing tick populations are still absent, representing another important gap for future research.

## CRediT authorship contribution statement

**Parisa Soltan-Alinejad:** Writing – review & editing, Writing – original draft, Investigation, Data curation, Conceptualization. **Mahmood Nikbakhtzadeh:** Writing – review & editing, Visualization, Validation. **Eslam Moradi-Asl:** Writing – review & editing, Writing – original draft, Software, Methodology.

## Ethical approval

The study was conducted by the ethical principles and the national norms and standards for conducting Medical Research in Iran. The study was approved by the Iran National Committee for Ethics in Biomedical Research (Approval ID: IR. ARUMS.REC.1403.483).

## Funding

This research was supported by 10.13039/501100006662Ardabil University of Medical Sciences (ARUMS), Ardabil, Iran, under Grant No.403000942.

## Conflict of interest statement

The authors declare that they have no conflict of interest.

## Data Availability

All data generated this study are available upon reasonable request to the corresponding author.
